# A Camera-Trap Home-Range Analysis of the Indian Leopard (*Panthera pardus fusca*) in Jaipur, India

**DOI:** 10.3390/ani10091600

**Published:** 2020-09-08

**Authors:** Swapnil Kumbhojkar, Reuven Yosef, Abhinav Mehta, Shrey Rakholia

**Affiliations:** 1 Jhalana Wildlife Research Foundation, Pune 411030, India; swapnil.kumbhojkar@gmail.com; 2 Eilat Campus, Ben Gurion University of the Negev, P. O. Box 272, Eilat 88106, Israel; 3 The Geographic Information System (TGIS) Laboratory, Sarkari Vasahat Road, Vastrapur, Ahmedabad 380052, India; infotgislab@gmail.com (A.M.); rakholias@gmail.com (S.R.)

**Keywords:** camera traps, home range, overlap, leopard, *Panthera pardus fusca*, Jhalana

## Abstract

**Simple Summary:**

Jhalana Reserve Forest (JRF) is engulfed by the city of Jaipur, India, with a human population of 3.9 million. Due to a continuum between human habitation and forests, frequent interactions with wildlife are inevitable. The leopard is the apex predator in JRF, sharing a habitat and resources with the human population. It is imperative to understand and study the leopard ecology to design and implement conservation strategies. We used a simple but effective and non-invasive technique—camera trapping—to identify leopards individually. The data analyzed using the minimum convex polygon (MCP) 100% method helped us estimate their home ranges. The use of camera traps was preferred over radio collaring, considering the permits of the Forest Department. We observed that males have larger home ranges than females. The females established their home ranges in the core area and on the fringes of the reserve. We discovered familial (mother and daughter) establishment of overlapping home ranges adjacent to one another. Three females who established themselves on the fringes were successful mothers raising cubs during the study period. The human population in the villages on the fringe areas share resources with the leopards, demonstrating that coexistence with predators is possible in a human-dominated landscape.

**Abstract:**

The suitability of the camera trap–retrap method was explored for identifying territories and studying the spatial distribution of leopards (*Panthera pardus fusca*) in the Jhalana Reserve Forest, Jaipur, India. Data from two years (November 2017 to November 2019, N = 23,208 trap-hours) were used to provide estimates of minimum home-range size and overlap. We conducted home-range analysis and estimation, using the minimum convex polygon (MCP) method with geographic information system (GIS) tools. We are aware of the limitations and advantages of camera trapping for long-term monitoring. However, the limitations of the research permit allowed only the use of camera traps to estimate the home ranges. A total of 25 leopards were identified (male = 8, female = 17). No territorial exclusivity was observed in either of the sexes. However, for seven females, we observed familial home-range overlaps wherein daughters established home ranges adjacent to or overlapping their natal areas. The median home range, as calculated from the MCP, was 305.9 ha for males and 170.3 ha for females. The median percentage overlap between males was 10.33%, while that between females was 3.97%. We concluded that camera trapping is an effective technique to map the territories of leopards, to document inter- and intraspecific behaviors, and to elucidate how familial relationships affect dispersal.

## 1. Introduction

One of the key components of a species’ ecology is its spatial organization. The minimum area required to sustain a viable population is determined by knowing the home range of individuals [1]. Burt [2] defined the home range as “the area inhabited by an individual for its normal activities such as mating, food gathering, and rearing of cubs”. The use of space is a key factor in animal ecology and behavior, which indicates the close relationship between animals and their habitat [3,4]. Typically, a home range contains all the resources essential for a species’ subsistence [5]. Home-range size is an important factor in the study of animals, as it is linked to various biological aspects such as behavior, physiology, abundance, and density [6,7,8].

Johansson et al. [9] suggested that a fundamental understanding of home-range size and potential overlap contributes to the effective conservation of populations of threatened species. This is particularly important in carnivores, whose substantial spatial needs and dietary requirements often conflict with human interests, creating a major challenge for biodiversity conservation and maintenance of a sustainable carnivore population [10]. This is further underscored when one needs to define conservation strategies for solitary felids and to understand their territorial structure [11].

Remotely operated cameras, equipped with motion sensors, are used to estimate populations of spotted or striped felids and to identify them individually. This has been demonstrated by scientists globally in their research on tigers (*Panthera tigris*) [12,13], jaguars (*P. onca*) [14,15,16,17], leopards (*P. pardus*) [18], snow leopards (*P. uncia*) [19], cheetahs (*Acinonyx jubatus*) [20], and ocelots (*Leopardus pardalis*) [21].

Gil-Sanchez et al. [22] suggested that the camera trap–retrap methodology is a useful tool in the estimation of home ranges and territory overlap for solitary carnivores. Furthermore, Maffei and Noss [23] observed that, due to the elusive and nocturnal behavior of carnivores, indirect methods like camera trapping and radio telemetry are effective for consistent studies. However, having compared the results of camera trapping to radio telemetry, they recommended camera trapping as a useful, non-invasive tool to predict the home ranges of target species.

Studies on snow leopards and pumas (*Puma concolor*) [24] demonstrated that males had larger home ranges than females. They further observed little variation in home-range size for females with a stable prey base. They contended that females use spacing to ensure access to an adequate supply of food (area minimizers), while males do it to ensure sexual access to females (area maximizers).

Others found that females establish their home ranges adjacent to their mothers, whereas males born in the same area tend to disperse [25,26]. They further observed that females occupy larger home ranges than required for subsistence and share a part of their territory with their daughters. The reproductive success potential is higher in maternal clans, whereas dispersal of males avoids inbreeding, as observed by these researchers. This was emphasized in a study that showed that historical exploitation of leopards in a well-protected reserve disrupted and reduced male dispersal, resulting in male natal philopatry, and ultimately leading to inbreeding [27].

Similarly, Sandell [28] observed that reproductive success is higher in females that use familiar home ranges, which are smaller in size and rich in resources, enabling subsistence in terms of food and shelter for themselves and their litter.

Home ranges for leopards in tropical forests are reportedly smaller compared to their counterparts in the savannah forest or dry deciduous forest [29,30,31,32]. Leopard home-range sizes can vary from 9 to 451 km^2^, and they are influenced by factors such as variation in habitat, prey abundance, mating opportunities, climatic conditions, and sharing of resources with sympatric carnivores such as striped hyena (*Hyaena hyaena*) [33,34,35,36,37]. Smaller home ranges indicate a higher biomass of available prey, resulting in a higher concentration of leopards per unit area [31].

The Indian/common leopard (*P. pardus fusca*) is classified as “vulnerable”, as per the International Union for Conservation of Nature (IUCN) red list (www.iucnredlist.org). Habitat loss and indiscriminate human persecution resulted in a decrease in its abundance and distribution, not only in India [38] but also in other parts of the world [39]. Coexistence (resource sharing) and separation (land sparing) are the two paragons that exemplify the challenge of carnivore conservation in a human-shared landscape [40].

The leopards of Jhalana Reserve Forest (JRF), in northwest India, have shared a habitat with and coexisted alongside the human population surrounding the reserve for an unknown number of centuries [41]. Understanding the home ranges of the leopards that coexist with human populations is critical for the conservation of their species and their continued persistence, as well as to ensure continued coexistence through a comprehensive understanding of the apex-predator ecology. Furthermore, because this is a breeding population wherein females are observed to regularly have two cubs bi-annually, we hoped to identify their home ranges and to examine the establishment of juveniles that also settle into the restricted area of 29 km^2^, instead of dispersing away from the JRF as previously observed. We used camera traps to estimate and understand the spatiotemporal organization of the leopards of Jhalana. The fixed-point camera traps generate limited point locality data for home-range estimation as compared to radio telemetry; however, it was proven to be an effective methodology for long-term monitoring [22]. Considering the size of the reserve, research permit restrictions, and the duration of study, we opted for the non-invasive and financially viable option of camera trapping.

The scarcity of resources (shared with villagers) in an island habitat and a healthy breeding population of leopards led us to hypothesize that, in Jhalana, a human-enclosed forest, leopards would have smaller home ranges with greater inter and intrasexual overlap. Moreover, the availability of domestic prey [41] in urban habitats would allow the leopards to utilize the human-dominated landscape for their subsistence.

## 2. Materials and Methods

### 2.1. Ethics Statement

Relevant permissions to carry out the research were obtained from the Office of the Chief Wildlife Warden, Rajasthan Forest Department (Research permit no. 2017/171). The study was conducted under the supervision of the Deputy Conservator of Forests (Wildlife), Jaipur, India.

### 2.2. Study Area

**Figure 1:**
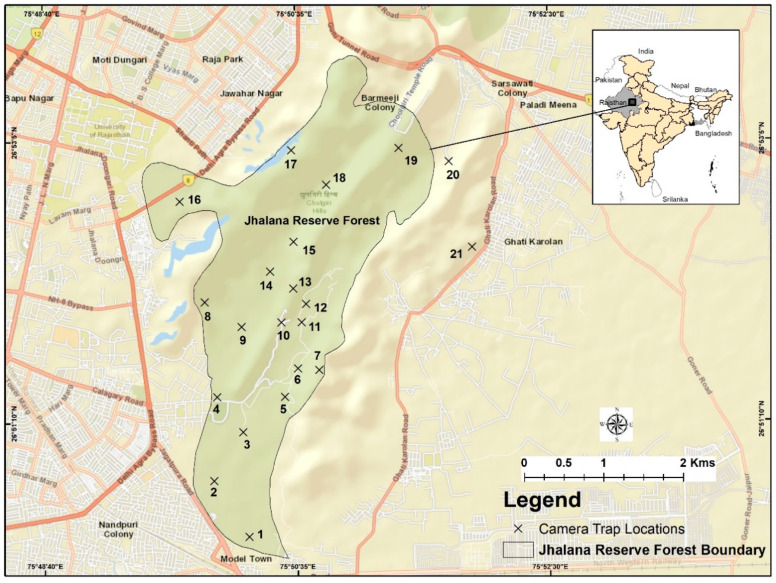
Map of the study area showing Jhalana Reserve Forest and its location along with the camera trap locations.

The study was conducted in JRF (26°51′ N 75°49′ E, 516 m AMSL) from November 2017 to November 2019 (Figure 1). JRF is characterized by a tropical dry deciduous forest and is located in the south-east corner of the city of Jaipur, India. It was designated as a Reserved Forest in 1961 following the provisions of the Rajasthan Forest Act of 1953, with a total area of 29 km^2^ with no separation of zones, as is customary in reserves/national parks, because of the unique situation wherein the forest has been completely encompassed by human habitation. During the 1980s, the main valley was planted with *Acacia tortilis* and *A. senegal*. Nearly all the ephemeral streams flow in south-west direction. Altitudes of the plains vary between 280 m in the south and 530 m in the north-east. Higher elevations in the north are in the form of low, flat-topped hills. JRF has no buffer or core areas and only a 2 m wall and a 3 m fence above it that separates the forested area from urban neighborhoods and villages. The wall is primarily constructed to restrict human intrusion into the reserve. Small openings in the fence permit the movement of wildlife in and out of the reserve, enabling them to use their traditional trails. The wall is no hinderance to the leopards’ movements to and from the urban areas. Tourists are allowed in jeep safaris on three routes. Due to the continuum of forests and human habitat and the fact that JRF was declared a reserve only in recent years, human intrusion inside the reserve is frequent, as is the spillover of wildlife into adjacent villages and urban habitats. However, in no case have we observed residents feeding the carnivores, or creating a feeding station.

### 2.3. Field Methods

Commercially manufactured Cuddeback (X-Change Color Model 1279, De Pere, WI, USA) cameras, with motion sensors, were used for this study. No bait or lure was used at any location to attract any of the wildlife [42]. Each camera was given a unique identification number and all the cameras recorded the date and time of the photograph. The locations of the trap positions were mapped using an independent GPS unit. Camera traps were deployed at 21 stations in an area of 29 km^2^ to cover the entire reserve and the fringe areas that are frequented by leopards as observed by us, the rangers and tour guides, and reports from the local villagers (Figure 1). Of 21 cameras used in all sessions, two were deployed on the fringe and 19 in the core area of the reserve. The duration of each camera trapping period was fixed at two weeks, i.e., 19 trapping events per year.

The standard time interval considered in a camera trapping survey is 30 min as subscribed by Cheyne et al. [43]. Other pilot studies [44] to calculate home-ranges from camera trap data used a time interval of 60 min. Some studies failed to mention this time interval [17,22]. Our time interval (10 min) is similar to that suggested by Pallemaerts et al. [1] in the study of home-ranges for Bornean clouded leopard *Neofelis diardi borneensis*. The time interval of 10 min, with a minimum of 15 s, gave us an opportunity of multiple photo capture events (one event being defined as one set of photos captured) while the leopard was still at the waterhole. This increased the probability of acquiring photos of facial markings and both the flanks to facilitate individual identification.

**Figure 2:**
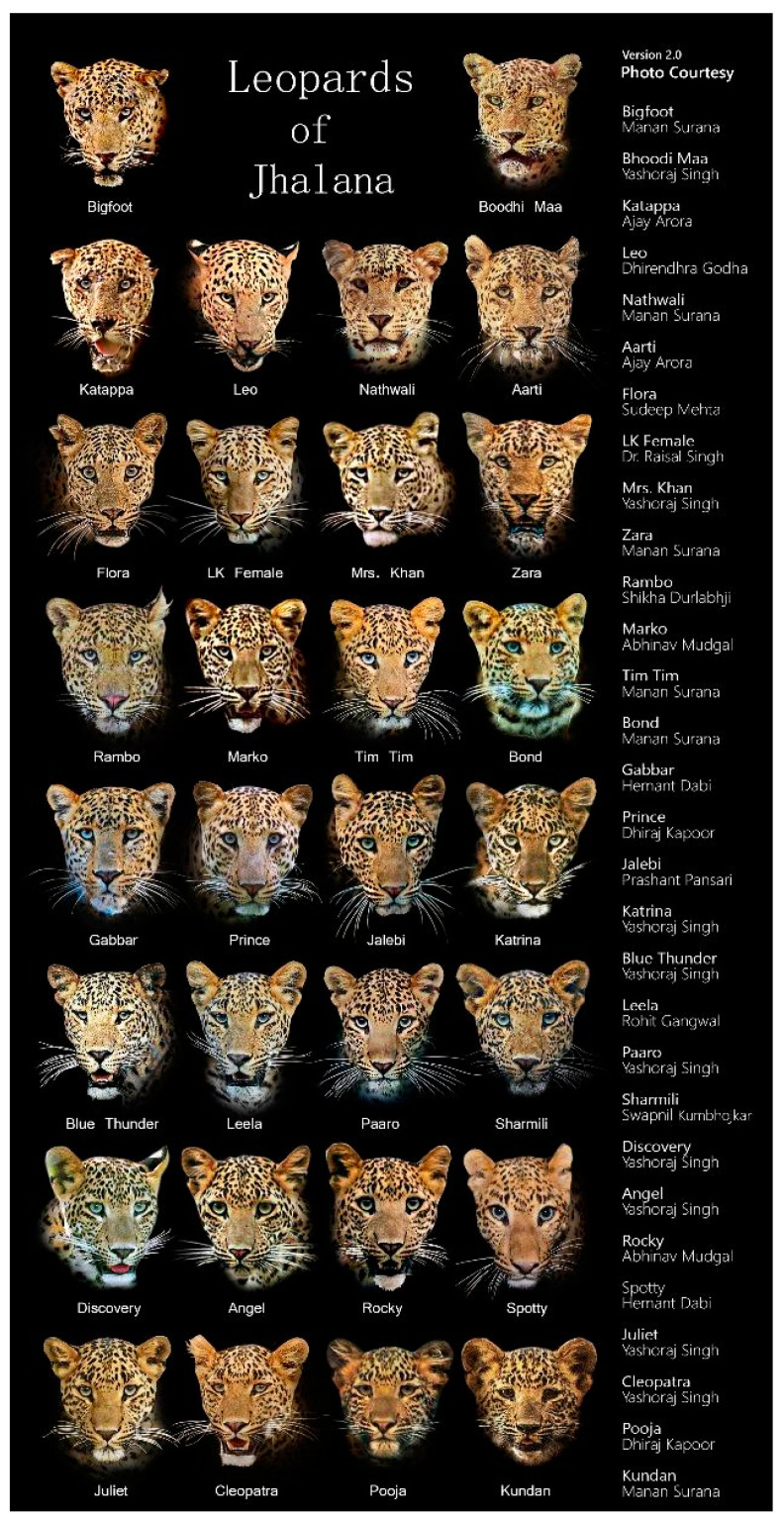
Facial portraits of the Leopards of Jhalana Forest Reserve during 2019. Text on the right indicates photo credits.

In earlier studies of tigers [12], leopards [45,46,47,48], leopard cat *Prionailurus bengalensis* [49], and dhole *Cuon alpinus* [44], researchers positioned a pair of cameras at each station to photograph both flanks of the target species for individual identification purposes. Similarly, our camera stations were selected based on the trails frequently traversed by the target species to facilitate individual identification based on facial markings and markings on the flanks (Figure 2). We considered individual identification (previously identified by local naturalists and safari guides) to be important in order to try and understand individual idiosyncrasies and to avoid overestimation of the study population, as demonstrated by Johansson et al. [50].

Owing to the absence of perennial water sources, the wildlife in JRF concentrates on the artificial waterholes created by the Rajasthan Forest Department. Considering the resources and facilities available at JRF, cameras were placed at these waterholes, knowing the limitations of the data collated in an optimal-stratified manner. Cameras (N = 2) on the fringe of the reserve were turned off and removed during the day and re-deployed at night to avoid theft or damage, whereas those inside the reserve remained untouched. Cameras were placed at a height of 45 to 50 cm above ground level covering the approach trails to the waterholes. In the fringe areas, camera traps were enclosed in boxes securely fitted to iron poles to ensure safety. The cameras on the fringe were placed at the waterholes artificially created by local villagers.

**Figure 3:**
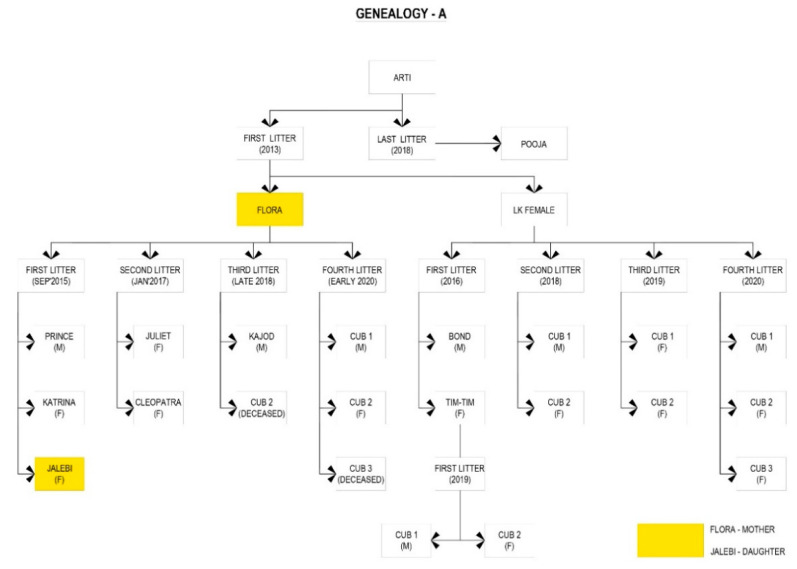

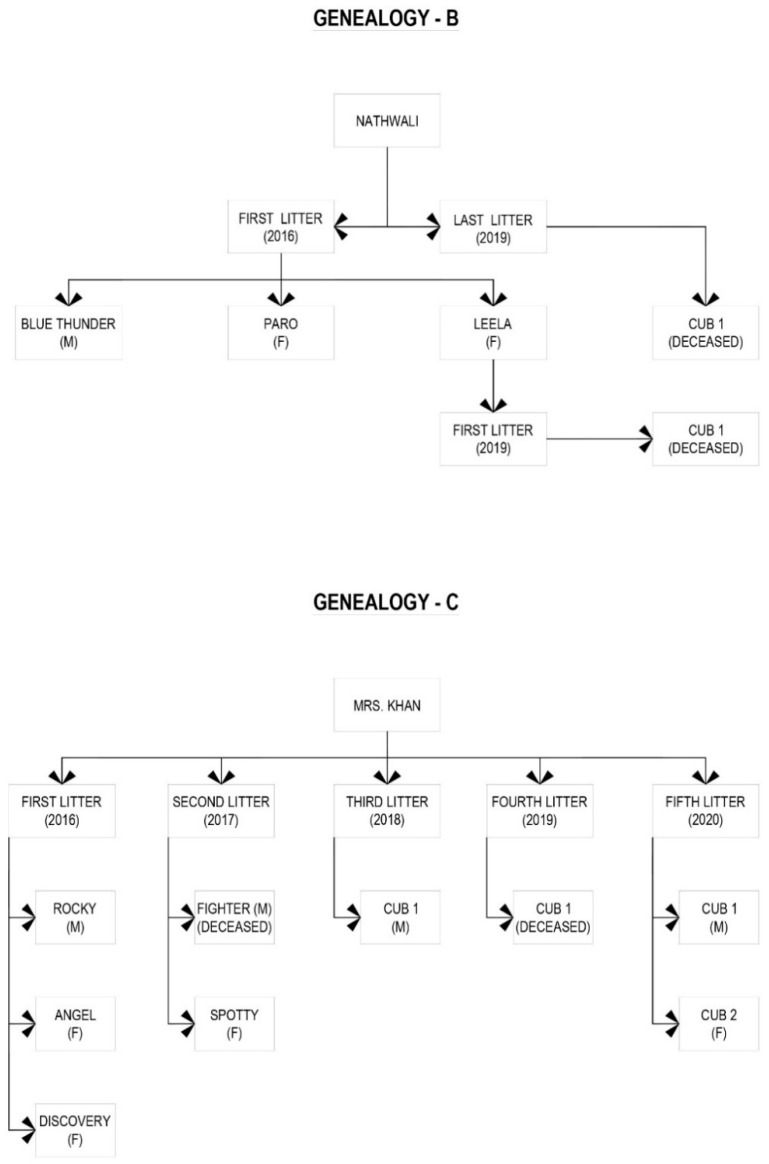
Genealogy of the three, dominant female leopards ((**A**)—Arti, (**B**)—Nathwali, (**C**)—Mrs Khan) of Jhalana Forest Reserve. Data correct for June 2020.

To document inter- and intra-specific behaviors, and to understand the familial-geographic spread of the leopards in the study area (Figure 3), the study was conducted for two successive years. Generally, camera trap surveys for the study of carnivore populations are conducted for short periods, as demonstrated by Karanth and Nichols [12] and Athreya et al. [46]. However, due to the paucity of data in JRF and limitations on sampling occasions due to various factors, such as thick undergrowth during the rainy season, disturbance due to human activities on the fringe, increased tourist inflow during festivals and holidays, this survey was conducted for two consecutive years and continues at present in a limited manner.

### 2.4. Data Analyses

Individual leopards were identified using facial markings and unique coat patterns on the right and left flanks of the body. All leopards were subsequently named for convenience by the tourist guides and forest rangers (Figure 2). Independent photo captures were defined by two paradigms: (1) the same individual captured at different locations at any given day and time, and (2) the same individual captured at the same location with an interval of minimum 15 min between two captures.

The captured point data can be analyzed and visualized using geographic information system (GIS) tools, especially in terms of home-range studies. GIS also has the capability of overlaying multiple layers of data (e.g., points, convex polygons etc.), which helps us to understand the movements and distribution of different species and thus pose an advantage over older traditional techniques [51].

The minimum convex polygon method (MCP) is a nonparametric method that estimates the home ranges based on the locations of all the observed positions by creating the smallest convex polygon [52], and is widely used for home-range analysis [53,54]. Home-range analysis and mapping were conducted based on observation points in GPS coordinates (i.e., latitude, longitude).

Another method employed in home-range studies is the fixed kernel density method (KDE). In order to compare between the two methods, we also conducted KDE and included 50% of the data points that were closest to each other. However, we considered it inadvisable to base our conclusions on KDE because the method requires large sampling points with an even distribution, as opposed to our data which are based on optimal-stratified sampling and were heavily clustered [55,56]. Male and female MCPs were overlaid using GIS tools (ESRI’s ArcGIS 10.6). The intersections between the MCPs determined the percent overlap. In this manner, the MCP area along with the percent overlap for each leopard was calculated. For further analysis and comparison, heatmap and box plots with *t*-test derived *p*-values were generated in R statistical software (R version 3.6.1).

Standard statistical data are expressed average (±SD). *t*-test was used for comparisons between parameters and *p* = 0.05 as the minimum acceptable level of significance.

## 3. Results

**Figure 4:**
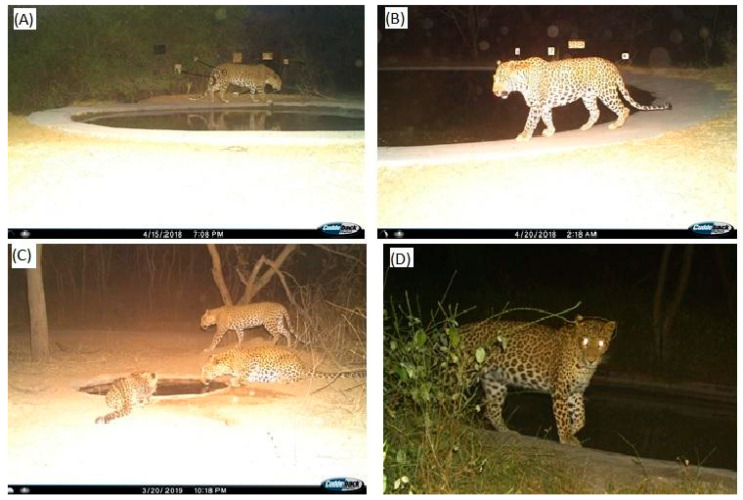
Camera trap images showing the sharing of resources and rosette/facial markings used to identify individuals at Jhalana Reserve Forest, India. The marked patterns on the flanks (Right flank (**A)**, Left flank (**B**)) are an example of the method used to identify an individual (Rambo, male).; (**C**) Adult female Jalebi sharing a waterhole with her mother Flora and her cub from a subsequent litter; (**D**) Facial marking of female Jalebi used for identification.

During 23,208 trap hours, a total of 16,327 photo-exposures were taken at an average of 0.7 photos/hour. Unclear or distorted pictures (N = 89; 0.55%) were omitted from our analyses. From the 661 (4.1%) photographs of leopards recorded during the study period (0.9 photos/day), we identified 25 individual leopards (M = 8, F = 17; Table 1), combining distinctive rosette patterns on both the flanks and facial markings from the photographs (Figure 4A,B,D). Three females were photographed along with their cubs.

### 3.1. Home Ranges and Core Areas of Male Leopards

**Figure 5:**
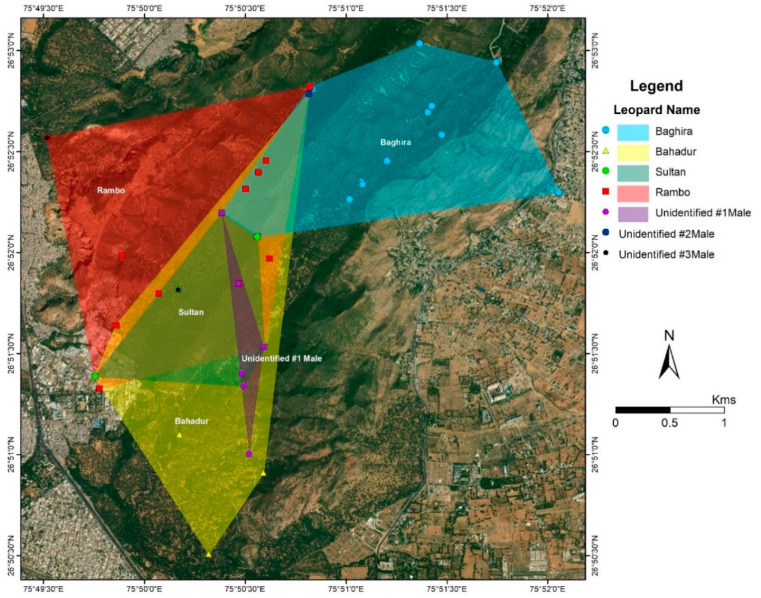
Home-ranges (MCPs) of male leopards of Jhalana Reserve Forest, India.

**Figure 6:**
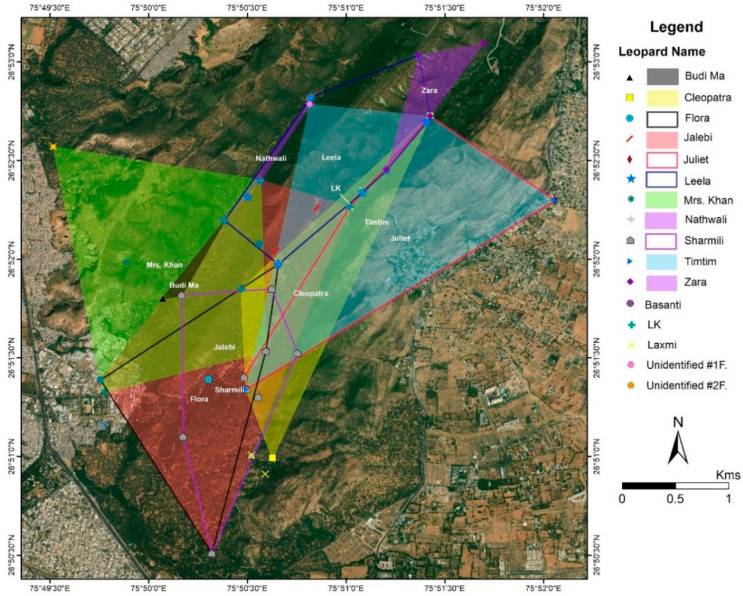
Home-ranges (MCPs) of female leopards of Jhalana Reserve Forest, India.

Of the 25 individually identified leopards, we were able to establish by camera-trap evidence and our frequent observations that four of the males (Figure 5) and eight of the females have established home-ranges in JRF (Figure 6). We inferred that three females and two males were floaters who passed through the area but did not remain for an extended period. The others were seven females and one male cub that accompanied their mothers and were also photographed. These individuals were spotted sporadically and hence their home ranges could not be identified. However, they were photographed in the same locations and appear to have shared similar home ranges.

The average home-range for the males was observed to be 2.45 km^2^ (±SD 1.63). The home-ranges for four out of the five male leopards were Sultan (1.28 km^2^), Baghira (3.06 km^2^), Bahadur (3.49 km^2^), and Rambo (4.18 km^2^; Figure 5). One unidentified male was observed intermittently and its home range could not be identified. Though no clear home-range exclusivity was observed for the males, Rambo predominantly occupied the western range and the core area, whereas, Bahadur, Sultan and the unidentified male occupied the core area. Baghira’s home-range extended from the north towards the north-east section of the reserve. The unidentified male was observed in the winter of 2018–2019, but not after March 2019.

**Figure 7:**
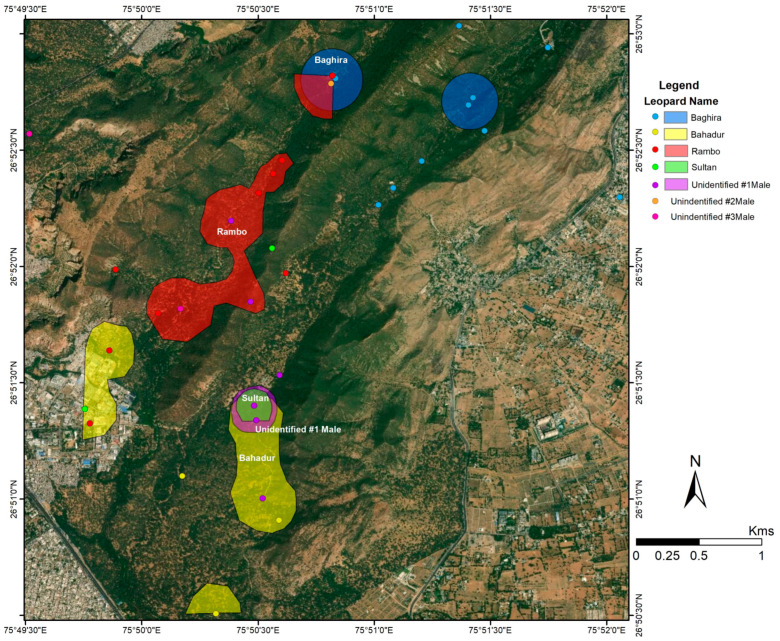
Kernel density analyses of 50% of clustered camera-trap photos of male leopards in Jhalana Forest Reserve.

The KDEs of the males were significantly smaller core areas (0.402 km^2^ (0.33)) than the home ranges (Figure 7). The analysis shows that Rambo and Bahadur appear to be the dominant males with wide spread distribution, while Baghira and Sultan are very restricted in their core areas.

### 3.2. Home Ranges and Core Areas of Female Leopards

The average home-range for the females was 1.89 km^2^ (±1.16 SD). Out of the 17 females identified, we were able to establish the home-ranges for eight—Nathwali (0.05 km^2^); Zara (0.29 km^2^); Sharmili (1.51 km^2^); Leela (1.58 km^2^); Flora (1.82 km^2^); Mrs. Khan (3.06 km^2^); Tim Tim (3.36 km^2^) and Jalebi (3.39 km^2^) (Figure 6). Jalebi occupies the largest home-range in the core area adjacent to that of her mother Flora (Figure 6). Nathwali occupies the smallest of the home-ranges (0.05 km^2^) on the western boundary of the reserve.

**Figure 8:**
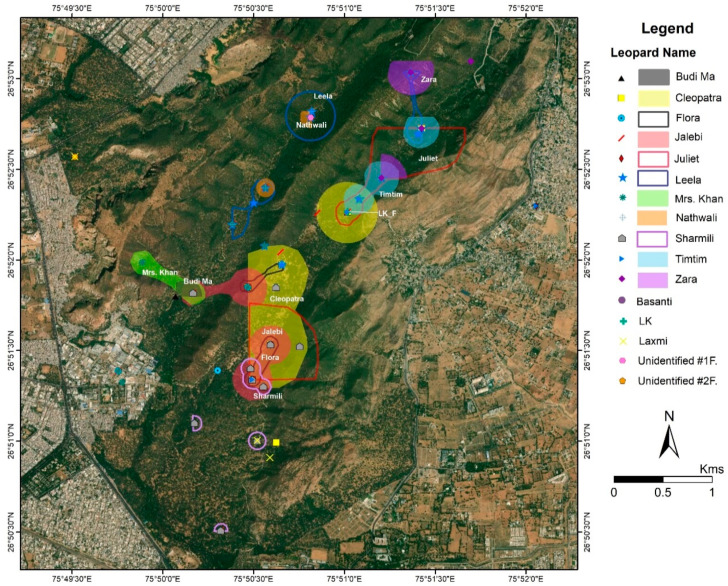
Kernel density analyses of 50% of clustered camera-trap photos of the female leopards in Jhalana Forest Reserve.

The core areas (KDE) of most of the females were significantly smaller (0.376 km^2^ ± 0.38) than their home ranges (MCP; Table 1). The exceptions were Tim Tim (1st litter of LK) and Jalebi (1st litter of Flora) who appear to roam freely between their natal home ranges and their own areas (Figure 8). However, similar to the home ranges (Figure 6), breeding females also display overlaps between core areas.

**Figure 9:**
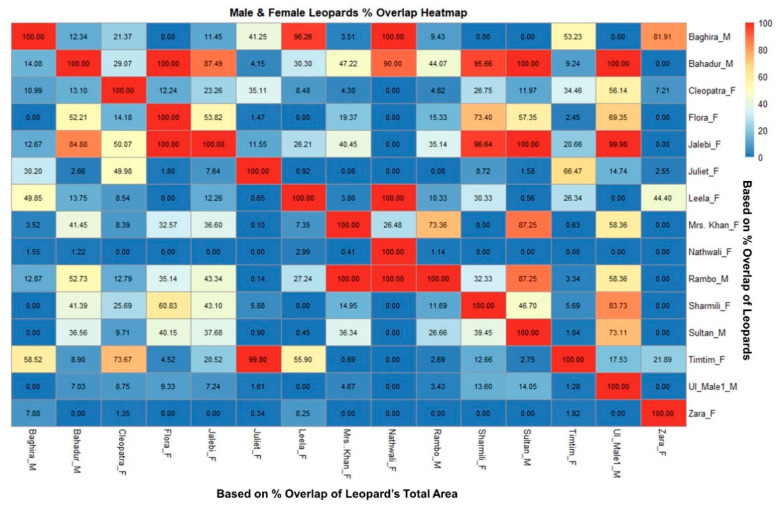
Heatmap matrix showing the percent overlap between the leopards of Jhalana Reserve Forest, India, along the *y*-axis.

Home-ranges of the males and those of the females overlapped considerably (Figure 9). Baghira completely covers the home-ranges of Nathwali (100%) and Leela (96%). Bahadur and Sultan appear to have overlapping home-ranges but differ also in the female home-ranges that they cover. Bahadur’s home-range overlaps with Flora (100%), Sharmili (96%), Nathwali (90%), Mrs. Khan (90%), and Jalebi (87%). Sultan overlapped to lesser degrees with Flora (40%), Sharmili (39%), Jalebi (38%), and Mrs Khan (37%). Rambo had a substantial home-range overlap with Nathwali and Mrs. Khan (100%), but also with Jalebi (43%) and Flora (35%).

**Figure 10:**
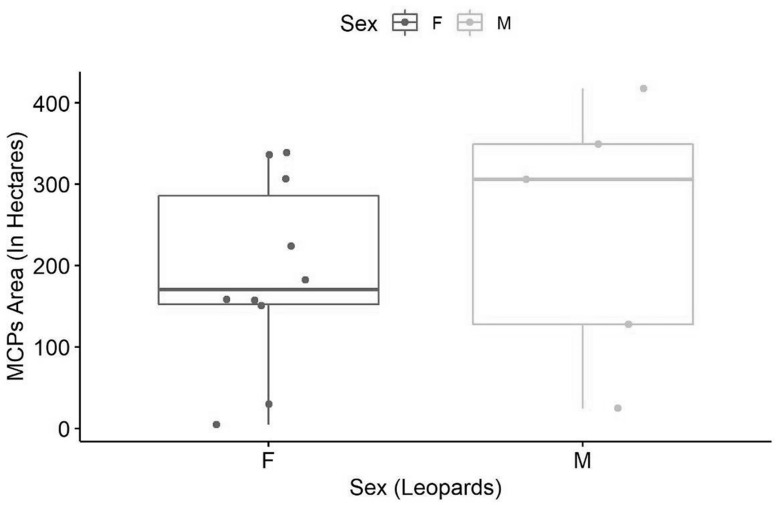
Home-range (MCPs) area (in ha) of male and female leopards of Jhalana Reserve Forest, India.

**Figure 11:**
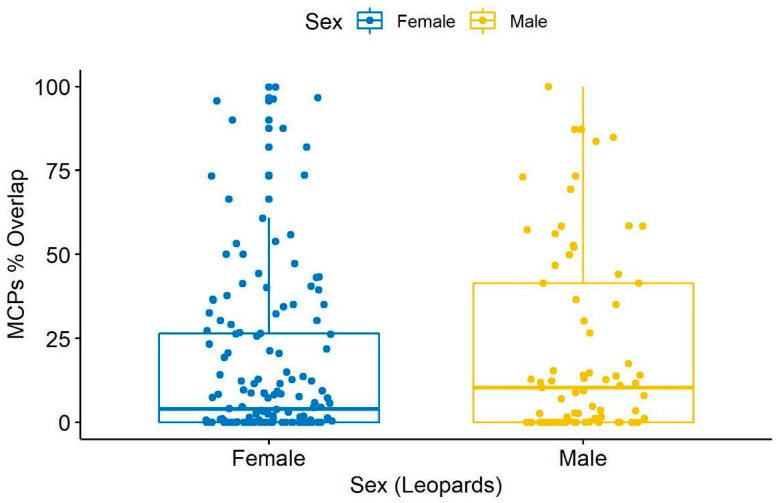
Percent Overlap of home-range (MCPs) area (in ha) of male and female leopards of Jhalana Reserve Forest, India.

There was no significant difference in home-range size (area) between both sexes i.e., male (2.45 ± SD) and female (1.89 ± SD; *p* = 0.52) (Figure 10). The average percent overlap for male leopards was 34.9% (±17.21 SD), higher than the average percent overlap for female leopards which was 25.98% (±SD 12.93) (Figure 11). However, the median home-range area of males (305.9 ha) is larger than that of the females (170.3 ha), and the median percent overlap in males (10.33%) is higher than that of females (3.97%; Figure 5 and Figure 6).

**Figure 12:**
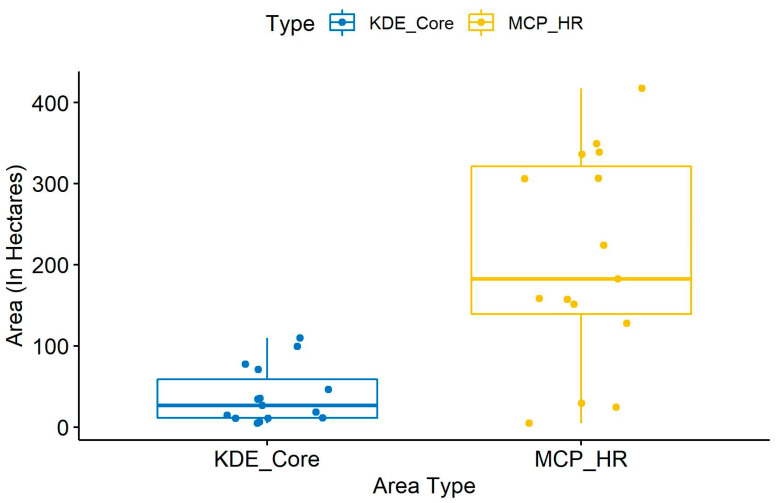
A comparison between KDE-derived core areas and MCP-derived home range areas show statistically significant differences (*p* = 0.00018) between the core areas and home ranges of the male and female leopards.

A comparison between KDE-derived core areas and MCP-derived home range areas shows that the differences are statistically significant (*p* = 0.00018) between the core areas and home ranges of the males and females (Figure 12). As mentioned earlier, KDE estimates can be highly biased if the location data are not evenly distributed and if the sample size is small (cf. 40). As a result, the KDE method of estimation appears to be unsuitable for home range estimation; however it allows us to depict core areas for each individual leopard in order to compare with the MCP home ranges.

For estimating a home range with the 100% MCP method, a minimum of three capture points (vertices) are required provided they are distributed on separate coordinates in space [54]. If multiple detections of a leopard occur on a single capture point, then home range cannot also be estimated as even for the simplest polygon, a minimum of three vertices are required (Table 1). Hence, we have not estimated the home ranges for three leopards (Budi Ma, Basanti and unidentified female F2) in spite of their having three capture points each. In a study of bearded saki monkeys (*Chiropotes satanas chiropotes*), Boyle et al. [54] found that MCP was more accurate in the calculation of home ranges than other methods compared.

**Figure 13:**
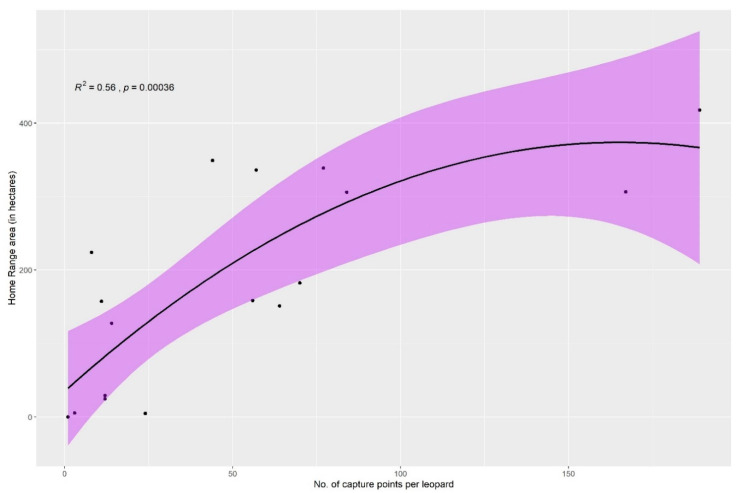
Saturation curve of home ranges of leopards (solid line) with respect to the number of capture points per leopard with 95% confidence interval (shaded area).

We found that as the number of capture points increased (as well as spatial distribution), the home range area also increased (Figure 13). The plotted points represent the actual home range size of the leopards, whereas the solid line is a smooth linear model based on the actual home range size and the data (capture) points in order to understand the overall trend. The individual-based rarefaction curve showed that for 1–2 capture points the home range size was 0 and the home range area estimates only showed a positive value when there were a minimum of three capture points. The increase in the cumulative number of home ranges was rapid for the first c. 80 data points but began to level off thereafter.

### 3.3. Familial Overlap

Flora, Sharmili, and Leela were observed to dominate the core tourist zone. Tim-Tim and Zara occupied a belt to the northeast, and Mrs. Khan established a home-range in the mines area to the Southwest (Figure 6). Two sub-adult females, Juliet and Cleopatra (litter of Flora from 2017), were photographed in an area adjacent to Flora; however, we have not considered their home-range estimates in this study due to their disappearance in April 2019.

Flora, one of the dominant females, was observed to shift her home-range boundaries towards the south-east of the reserve in 2019 and to accommodate the establishment of her daughter Jalebi from the litter of 2015. Similarly, Nathwali, who is the mother of Leela, allowed her to settle adjacent to her home-range and with considerable overlap. Similarly, LK accommodated her daughter Tim-Tim in the Bhomiyaji area to the Northeast of the core area (pers. obs.).

## 4. Discussion

In the first ever study of the home-ranges of the leopards of JRF, we used a camera trapping technique to identify individual leopards and to also estimate their home-ranges. The importance of this study is that in spite of many publications on the study species in India, only one has studied home range/territory size for leopards. We were also able to elucidate how familial relationships influence decision making in adult females to allow their daughters to establish home-ranges adjacent to their natal areas. Females in polygynous mammalian, especially carnivore, species are known to be philopatric and abdicate part of their home range to form matrilineal kin clusters [27]. Studies on leopards in Namibia and Israel demonstrated that sub adult males disperse, while the sub-adult females, in independence, form home ranges adjacent to their mothers with a higher degree of overlap and benefit from local knowledge of available resources [57,58,59]. This has also been demonstrated for snow leopards in Kyrgyztan, where eight females of a local population were directly related to each other [26]. Similarly, we identified three home-range accommodations and overlap between females and their subadult cubs—Flora with Jalebi her daughter from 2015, Nathwali with Leela, and LK with Tim-Tim. Similar behavior was observed in African wild dog *Lycaon pictus* packs [60], wherein dispersing individuals established territories adjacent to their relatives with substantial and regular overlap. Wolf and Trillmich [61] stated that natal philopatry is a key paradigm to promote definitive genetic population structures and to ensure continuity. This leads to “budding” (a pattern where dispersers establish their home-ranges close to their birthplace), which further results in kin-clustering (Figure 4C). This is demonstrated in our study by the home-range overlap of Flora and Jalebi, LK and Tim-Tim, and Nathwali and Leela. The southeast corner of JRF was previously occupied by another female, Arti (deceased in 2019), the mother of Flora and LK. It is of interest that Flora and LK are sisters from the same litter in 2013 and initially had neighboring home-ranges but have now shifted away from each other, allowing their respective daughters to establish home-ranges between them. We assume that apart from kin-clustering, another reason could be the frequent confrontations with other sympatric carnivores, especially striped hyena.

Spatial kinship patterns in solitary felids was shown to be affected by anthropogenic mortality and persecution [27]. Young male leopards had shorter dispersal distances and established home ranges adjacent to their mothers and/or sisters [62], increasing the probability of inbreeding. In our study, the spatial distribution of the females and their matrilineal kin clusters, and the assumed dispersal of other female and male cubs away from the study area, clearly demonstrate the porosity of the Jhalana leopard population. This assumption is further supported by records of the five floaters and their sporadic appearances with no established home ranges. Our findings support Sandell [28], who suggested that floaters/roamers are non-territorial males that increase their range during mating season to compete over receptive females.

We further assume that the leopard population of Jhalana is a “source” population [63] and have survived in spite of anthropogenic disturbance (e.g., ecotourism), illegal intrusion of the villagers (grazing, wood-cutting, etc.), and in the last three years extensive construction work in the form of building the perimeter wall and laying infrastructure for water and CCTV surveillance towers. Our findings merit further studies of the gene flow in the population and the wildlife corridors used by the leopards for dispersal and by floaters.

We believe that a study over an extended period of time would show further mother–daughter range accommodations. Zara and LK occupied home-ranges in the northeast, and Mrs. Khan in the south-west of the reserve throughout the study period. Mrs. Khan gave birth to a male cub in the first litter (2018) and a male cub (2019) in the second litter (Figure 3). LK raised two cubs (one male, one female) in 2018 and two females in 2019. The south-west region is an abandoned granite mine and the north-east area is on the fringe of the reserve bordering the village of Bhomiyaji. Both areas have dense human populations sharing the habitat and resources with the wildlife of JRF.

Furthermore, the grouping of leopards is unusual but has been observed in solitary species such as snow leopards in Bhutan [64], when three individuals were photographed five times at the same camera station in a period of four months. A congregation of seven individuals was also recorded in another camera trap study [65]. Similar behavior was demonstrated by Flora, photographed with Jalebi, her daughter from 2015, and Kajod, a subadult male from the 2019 litter (Figure 4C).

Regarding the home-ranges of females, Flora (1.82 km^2^) and Mrs. Khan (3.06 km^2^), rearing cubs every year, have smaller home-ranges as compared to Jalebi (3.39 km^2^), who has not yet had a litter during the study period. These results concur with previous studies for leopards [57,58,59] and tigers [66] which observed that the movements of mothers rearing cubs are restricted to avoid infanticide, especially from conspecific males. However, we recommend further investigation to confirm this behavior in JFR, and to check if there are sex-specific, prey-density related seasonal fluctuations of home range sizes as suggested [24].

Researchers found that in snow leopards the estimated home range varied between 11 and 37 km^2^ [67,68,69,70]. The home ranges of leopards ranged from 8 to 13 km^2^ in Chitwan National Park, 14.8 km^2^ in Kruger National Park in Southern Africa and in Kenya, 33 to 62 km^2^ in the Russian Far East, and 128 to 487 km^2^ in the Kalahari Desert [71]. The overlap analysis based on home-range plays an important role in determining the spatial characteristics and territoriality of species specifically when topographic and social factors are considered [72]. However, in our study, MCP and KDE analyses demonstrated that males had larger home-ranges than females, and intrasexual overlap was comparatively low in both sexes (Figure 9). Though some studies [68,73,74,75] did not record major differences in the home-range size of African male and female leopards, most other studies on leopards in the Middle East and Southern Africa [59,76,77,78] and snow leopards [9] revealed that adult males have larger home-ranges than conspecific females. Johansson et al. [24] showed that male and female territories intersected and hinted at female choice and male mate-guarding.

A study on the diet composition of the leopards of JRF [41], similar to other studies conducted on the leopards of western Himalayan region [79], clearly indicated the dependence of leopards on domesticated animals such as feral dogs, cats, domestic pigs and cattle which are predated in urban neighborhoods adjacent to the reserve. This may also explain why we have documented smaller home-ranges for the individuals that live in the peripheral areas of JRF– it may be either because they have a large prey base at their disposal in the urban areas or that most of their home-range spills over into the villages and neighborhoods which we have not monitored.

Karanth and Sunquist [80] mentioned low capture probability for tiger cubs that are less than a year old. However, we observed that Flora and her cubs became accustomed to the presence of the cameras (Figure 4C). Flora, one of the dominant females, occupying the core area in JRF, was photographed with two female cubs from her first litter in 2018 and one male cub from her second litter in 2019. Similarly, Mrs. Khan was photographed with one male cub in her first litter (2018) and one female cub in her second litter (2019), and so was Tim-Tim (one male and one female cub from 2019 litter). The females Flora, Mrs. Khan and LK from Jhalana exhibit this characteristic with successive successful litters as recorded during our study period.

In spite of many camera-trap or radio-collar studies, our study is the first to show a cumulative curve for home range size in leopards. Home ranges can be estimated beginning from a minimum of three geographically spread out data points and certainty increases with a greater number of such points. In all our territories, we only calculated home ranges for those individuals with a minimum of 10 different locations (Table 1). The saturation curve suggests that ca. 80 locations are enough to know the maximum home range in leopards (Figure 13). This is important for long-term studies that wish to optimize effort and time invested in the research.

Although radio-telemetry is an accurate and effective method to estimate home ranges of free ranging solitary felids, especially where the home ranges and size of the study area are large [57,75], and we understood the limitations of camera trapping and that it generates data only from fixed point camera stations, we were limited by the research permit issued by the Rajasthan Forest Department to no handling of animals or telemetry. Jhalana, with an area of 29 km^2^, hosts a leopard population of 25 individuals. The individual leopards (females and males) were photographed consistently only at certain camera stations throughout the study period of two years (Table 2). Furthermore, these data indicate that the individuals have consistently utilized and exploited the area and its resources where they are photographed. Our estimation of the home ranges is based on the temporal distribution of individuals over a specific area as recorded by the camera stations. We assume that the estimated home ranges are accurate, considering the fact that the camera stations were placed to cover the entire reserve (Figure 1) and these individuals were never photographed outside their estimated home ranges. Furthermore, our findings concur with those of earlier studies [22,23] of home range estimation using camera trap methodology. Our study merits the use of radio-telemetry for further verification of home range sizes even though radio-telemetry may be more accurate.

The above gives a different perspective of the leopards and where the conservation agencies and authorities can focus on integrating wildlife into human-modified landscapes and enhancing an understanding and tolerance of humans towards the predators and vice versa [81]. Athreya et al. [46] suggested that the requirements of the villagers and their daily needs must be addressed to enhance their coexistence with the wildlife. We agree, but in light of our findings, we consider the conclusions of Stein et al. [57], who studied leopards in Namibia, to be pertinent and that research cannot be limited to the boundaries of protected areas but also to incorporate adjacent areas. This is especially important in JRF, wherein there is no buffer zone and only a 5 m fence separating the carnivores from the city of Jaipur and other surrounding villages.

Jhalana proves to be a very interesting and complex forest for the study of the Indian leopard. In Nepal, leopard density was estimated to range between 1.5/100 km^2^ in the Terai region [82] and 3.31 and 3.45/100 km^2^ in the Chitwan National Park [83,84], and 3.48/100 km^2^ in Parsa National Park [85]. Athreya et al. [46] reported a density of 4.8/100 km^2^ in Maharashtra, India. In JFR, we have 25 adult leopards living in a 29 km^2^ area, i.e., 0.86 leopards/km^2^ which allows for the highest density of Leopards known anywhere in the world.

## 5. Conclusions

In conclusion, we found that the monitoring program and home-range estimate using camera traps is beneficial for understanding ecological and behavioral aspects of carnivores and for conservation planning and management. We also established that populations of threatened felid species with restricted ranges can be studied using long-term passive and non-intrusive monitoring. We recommend the Rajasthan Forest Department to undertake an intensive public outreach program in all the neighborhoods and villages bordering the forest to ensure the continued coexistence between the humans of Jaipur and the leopards of Jhalana.

## Figures and Tables

**Table 1:** List of the identified leopards of Jhalana Reserve Forest, indicating the number of photographs used/discarded for identification and estimation of home ranges (minimum convex polygon method, MCP) and core areas (fixed kernel density method, KDE). NI denotes “Not identified”.

Sr. No.	Leopard	Sex	No. of Photo-Captures/Individual	No. of Photo-Captures Discarded/Individual Due to Repetition	Home Range (MCP) km^2^	Core Range (KDE) km^2^
1	Sultan	M	11	3	1.28	0.062
2	Baghira	M	61	23	3.06	0.355
3	Bahadur	M	33	11	3.49	0.772
4	Rambo	M	116	46	4.18	0.712
5	Unidentified male_1	M	10	2	0.25	0.111
6	Nathwali	F	18	6	0.05	0.048
7	Zara	F	11	1	0.29	0.185
8	Sharmili	F	46	18	1.51	0.109
9	Leela	F	43	13	1.58	0.341
10	Flora	F	51	19	1.82	0.106
11	Mrs Khan	F	103	64	3.06	0.146
12	Tim tim	F	35	22	3.36	3.36
13	Jalebi	F	51	26	3.39	3.39
14	Juliet	F	8	0	2.24	0.995
15	Cleopatra	F	11	0	1.57	1.097
16	LK Female	F	4	0	NI	NI
17	Basanti	F	3	0	NI	NI
18	Unidentified F1	F	1	0	NI	NI
19	Unidentified F2	F	3	0	NI	NI
20	Laxmi	F	7	0	NI	NI
21	Budi Ma	F	3	0	NI	NI
22	Tim tim cub	F	15	0	3.36	0.268
23	Unidentified M2	M	1	0	NI	NI
24	Unidentified M3	M	5	0	NI	NI
25	Flora cub	M	11	0	NI	NI

**Table 2:** Percentage of photographs at camera stations for the 11 leopards whose home ranges are estimated with a confidence of reliability. (N = Camera Station Number, UM = Unidentified Male 1).

N	Sultan	Baghira	Bahadur	Rambo	UM	Nathwali	Zara	Sharmili	Leela	Flora	Mrs Khan	Tim Tim	Jalebi
1			47					8		5			5
2													
3			2		8			8		3			
4													
5	64		13		50			72		22			26
6	7			3	8			3		53	2		25
7								3					
8				1							38		
9	7		26	16				3		3	48		7
10			4	2	8			2		7	1		20
11										2			4
12		3		1					4	5			
13	7	4							19		2		6
14	7	3	2	20	25	4			10		6		3
15	7	2		3		20			7		3		4
16													
17				4									
18		42	2	49		75	17		42			5	
19							33		7			54	
20		12					50		9			38	
21		28										3	
